# Biobased Thermoplastic Elastomers: Structure-Property Relationship of Poly(hexamethylene 2,5-furanodicarboxylate)-Block-Poly(tetrahydrofuran) Copolymers Prepared by Melt Polycondensation

**DOI:** 10.3390/polym13030397

**Published:** 2021-01-27

**Authors:** Sandra Paszkiewicz, Izabela Irska, Agata Zubkiewicz, Anna Szymczyk, Elżbieta Piesowicz, Zbigniew Rozwadowski, Krzysztof Goracy

**Affiliations:** 1 Department of Materials Technologies, West Pomeranian University of Technology, Al. Piastów 19, 70-310 Szczecin, Poland; izabela.irska@zut.edu.pl (I.I.); elzbieta.piesowicz@zut.edu.pl (E.P.); 2 Department of Technical Physics, West Pomeranian University of Technology, Al. Piastów 48, 70-311 Szczecin, Poland; agata.zubkiewicz@zut.edu.pl (A.Z.); anna.szymczyk@zut.edu.pl (A.S.); 3 Department of Inorganic and Analytical Chemistry, West Pomeranian University of Technology, Al. Piastów 42, 71-065 Szczecin, Poland; zbigniew.rozwadowski@zut.edu.pl; 4 Department of Polymers and Biomaterials Science, Nanotechnology Center for Research and Education, West Pomeranian University of Technology, Al. Piastów 45, 71-311 Szczecin, Poland; krzysztof.goracy@zut.edu.pl

**Keywords:** furan-based copolymers, biocopolyesters, poly(hexamethylene 2,5-furanodicarboxylate), bio-poly(tetrahydrofuran), melt polycondensation, phase structure, morphology, thermal properties, mechanical performance

## Abstract

A series of poly(hexamethylene 2,5-furanodicarboxylate)-block-poly(tetrahydrofuran) (PHF-*b*-F-pTHF) copolymers were synthesized using a two-stage procedure, employing transesterification and polycondensation. The content of pTHF flexible segments varied from 25 to 75 wt.%. ^1^H nuclear magnetic resonance (NMR) and Fourier transformed infrared spectroscopy (FTIR) analyses were applied to confirm the molecular structure of the materials. Differential scanning calorimetry (DSC), dynamic mechanical measurements (DMTA), and X-ray diffraction (XRD) allowed characterizing the supramolecular structure of the synthesized copolymers. SEM analysis was applied to show the differences in the block copolymers’ morphologies concerning their chemical structure. The influence of the number of flexible segments in the copolymers on the phase transition temperatures, thermal properties, as well as the thermo-oxidative and thermal stability was analyzed. TGA analysis, along with tensile tests (static and cyclic), confirmed the utilitarian performance of the synthesized bio-based materials. It was found that an increase in the amount of pTHF caused the increase of both number-average and weight-average molecular weights and intrinsic viscosities, and at the same time causing the shift of the values of phase transition temperatures toward lower ones. Besides, PHF-*b*-F-pTHF containing 75 wt.% of F-pTHF units was proved to be a promising thermoplastic shape memory polymer (SMP) with a switching temperature of 20 °C.

## 1. Introduction

Thermoplastic elastomers are widely used as soft materials in both industry and medicine. One can find multi-blocks segmented poly(ether-ester)s (PEE)s as engineering thermoplastic elastomers due to their attractive combination of strength, cold-temperature flexibility, high entropy elasticity, melt stability, high crystallization rates, and many more [1,2]. Such great attention from many scientific groups results from the combination of rubber-like properties in the solid-state and melt processability [3]. PEEs are composed of rigid segments with a high glass transition (T_g_) and a rubbery segment with low T_g_, whereas the domains of hard crystalline segments are interconnected by the soft matrix segments [1]. The most prominent representative of this type of materials is Hytrel^®^ (Dupont, Wilmington, DE, USA), a whole group of copolymers where the rigid segment is based on poly(butylene terephthalate) (PBT) and the rubbery segment is polytetrahydrofuran (pTHF), also called poly(tetramethylene oxide) (PTMO). However, for the next generation elastomers, environmentally friendly materials are needed [4]. To make it accomplish, several strategies included replacing petrochemicals with biobased and renewable raw materials, replacing conventional metal catalysts with environmentally friendly or renewable catalysts, and incorporating chemical recyclability and environmental biodegradability into the elastomers, have been applied. In 2015, for the first time, BASF has made available bio-based polyTHF with a molecular weight of 1000 g/mol (PolyTHF^®^1000) [5]. The bio-based polyTHF^®^1000, which is derived entirely from biomass feedstocks, is found to be identical in quality to the petrochemical-based product. The process hinges on a microbial fermentation of sugars to produce 1,4-butanediol (BDO), which is then purified and polymerized. PolyTHF is primarily used as a component of thermoplastic polyurethanes, poly(ether-esters), and poly(ether-amides) [6].

Polyesters based on 2,5-furan dicarboxylic acid (FDCA) or its ester derivatives (for instance, dimethyl 2,5-furan dicarboxylate, DMFDC) like poly(ethylene 2,5-furan dicarboxylate) (PEF), poly(propylene 2,5-furan dicarboxylate) (PPF), and poly(butylene 2,5-furan dicarboxylate) (PBF) can be successfully treated as the biobased alternatives to the terephthalic acid (TPA)–based polyesters, like poly(ethylene terephthalate) (PET), poly(propylene terephthalate) (PPT), and poly(butylene terephthalate) (PBT), which are produced and applied in a vast amount of commercial applications [7,8,9,10,11,12,13]. Therefore, even though polyesters based on furan derivatives have been known for years, the challenge remains to obtain high molecular weight materials without changing the color to brown-black, resulting from thermal decomposition of FDCA [14,15]. Almost a decade ago, Gandini et al. [11,16] reported the successful synthesis of high molecular weight polyesters and copolyesters based on FDCA and various diols prepared by straightforward routes, using mild conditions. It is of foremost importance that the structure of FDCA is quite similar to the one of TPA, however, one can produce FDCA from biomass or its derived sugars or platform chemicals, which generally involves chemical, biological, and electrochemical methods [17]. Therefore FDCA has been considered as a replacement for fossil-based TPA [18,19,20]. FDCA has a large potential as a bio-based monomer for the synthesis of polyesters, polyamides, polyurethanes, and their copolymers [7,16], and thus it is one of the most important building blocks or top value-added chemicals derived from biomass by the US Department of Energy [21,22].

Poly(hexamethylene terephthalate) (PHT) is, so far, a noncommercial aromatic polyester of both academic and applied interest [21,23,24,25], which, similarly to other aromatic polyesters, exhibits fair mechanical properties and an excellent chemical resistance [26]. Due to the presence of the flexible segment composed of six methylene groups in the polyester chain, PHT exhibits a relatively low melting temperature (Tm ≈ 140 °C), which one can find as advantageous for more economical and easier processing procedures [27]. In turn, a furanoate homolog of PHT is poly(hexamethylene 2,5-furan dicarboxylate) (PHF). Synthesis of PHF was first reported in 1978 by Moore and Kelly [28], while recently, a series of poly(alkylene 2,5- furan dicarboxylate)s including PHF were synthesized and characterized [21,29,30,31,32]. In turn, Zhang et al. [33] systematically summarized recent progress in the making of FDCA-based polyesters and their copolyesters (including PHF), especially highlighting the progress and fundamental aspects for their synthesis and properties. PHF is a polyester that is synthesized from monomers produced from renewable resources, especially concerning FDCA. But, since Rennovia announced the successfully operating core pilot plant processes for the cost-advantaged production of 1,6-hexanediol (1,6-HDO) from renewable feedstocks (in this case, sugars) [34,35], one can synthesize PHF entirely from renewable raw materials. 1,6-HDO, which is widely used today in a variety of formulated products, including coatings, adhesives, and elastomers, is anticipated to have greatly reduced greenhouse gas and environmental impacts versus petroleum-based 1,6-HDO. For instance, Zhang et al. [32] synthesized PHF from FDCA and 1,6-HDO via direct esterification. PHF characteristics are considered to be quite satisfactory as it exhibits comparable or higher thermal properties, but also higher mechanical properties, compared to several other aliphatic polyesters, such as poly(L-lactic acid) (PLLA) or poly(butylene succinate) (PBS) [29] or even PET or PEF [32]. For example, the PEF synthesized by Jiang et al. [29] with Mw of 66,700 g/mol exhibits a tensile modulus, tensile strength, and elongation at break of 493 MPa, 35.5 MPa, and 210%, respectively, and T_g_ and T_m_ of 28.1 and 148.2 °C, respectively. While, the PHF obtained by Zhang et al. [32] exhibited a typical (110) plane, (010) plane, and (111) plane at 2θ = 13.78° (d = 6.42 Å), 17.06° (d = 5.19 Å), and 24.9° (d = 3.58 Å) with an intrinsic viscosity of 0.803 dL/g, average Young’s modulus of 479 MPa, and maximum tensile strength of 36.5 MPa. In turn, Papageorgiou et al. [21], who studied the crystallization and thermal degradation behavior of PHF, found that the T_m_ and T_g_ of the obtained PHF were 145 °C and 7 °C, respectively, and that PHF exhibited multiple melting behavior after the crystallization process, which was mainly due to partial melting, recrystallization, and final melting. Therefore, it can be used in some applications, where up to this time point, fossil-based polyesters like terephthalates were most commonly used. However, due to, among others, the flammability, the applications of PHF, despite excellent mechanical properties, have been limited. Therefore, the copolyesters of PHF also attracted much attention [32,36,37]. For instance, Wang et al. [37] synthesized phosphorus-containing PHF (PHFCs) with good thermal performance, where T_g_ value decreased slightly along with the increasing molar content of 2-carboxyethyl (phenyl)phosphinic acid. Whereas, Wang et al. [36] prepared via the two-step melt polycondensation method PHF-b-poly(ethylene glycol) with excellent thermal stability with excellent shape-memory ability. In addition, Xie et al. [38], also via the melt polycondensation method, prepared poly(ethylene-co-hexamethylene 2,5-furandicarboxylate) (PEHF) copolyesters, in which, depending on the synthesis conditions and amount of PHF, the copolyesters were amorphous or semicrystalline. The synthesized copolyesters exhibited better thermal properties had a single T_g,_ which decreased along with the increasing content of PHF in the copolyester, and good mechanical performance, where the tensile modulus and tensile strength of the non-crystalline copolyester decreased. However, the elongation at break and impact strength increased along with the increase of the molar content of PHF. The tensile properties of PEHF copolyesters were similar to those of bottle grade PET.

To the best of our knowledge, for the first time, we will present the structure-property relationship of PHF-*b*-F-pTHF copoly(ether-esters) synthesized via a melt polycondensation process entirely from renewable raw materials. The composition and chemical structure of the series of block copolymers were determined by ^1^H quantitative nuclear magnetic resonance (NMR) and Fourier transform infrared spectroscopy (FTIR). The temperatures corresponding to phase transition changes were characterized by differential scanning calorimetry (DSC) and dynamic mechanical thermal analysis (DMTA). On the basis of preliminary DSC and DMTA tests, materials that can exhibit a thermally-activated shape memory behavior were preselected and investigated via cyclic thermo-mechanical analysis. The crystallization behavior of the materials was characterized by X-ray diffraction. The changes in the morphology resulting from the changes in the phase structure and composition were characterized using scanning electron microscopy (SEM). Additionally, the influence of the bio-based pTHF segment on the tensile properties (static and cyclic) and thermal and thermo-oxidative stability has been analyzed.

## 2. Materials and Methods

### 2.1. Synthesis of PHF-b-F-pTHF Copolymers

The series PHF-*b*-F-pTHF copolymers were prepared from renewable raw materials: dimethyl 2,5-furanodicarboxylate (DMFDC, 99%, Henan Coreychem Co., Ltd., Zhengzhou, China), 1,6-hexylene glycol (HDO, Rennovia Inc., Santa Clara, CA, USA), and poly(tetrahydrofuran) (pTHF, polyTHF^®^1000, Ludwigshafen, BASF, Germany) with a molecular weight of 1000 g/mol. The synthesis procedure consisted of two stages: (i) the first step involved the transesterification of DMFDC by HDO in the presence of the first portion of catalyst (tetrabuthyl orthotitanate, Ti(OBu)_4_, (Fluka)), and (ii) the second step which was polycondensation, where polyTHF^®^1000 was added in the presence of the second portion of catalyst (also Ti(OBu)_4_) and thermal stabilizer, Irganox 1010 (Ciba–Geigy, Basel, Switzerland). The reaction was carried out in a 1 dm^3^ high-pressure reactor (Autoclave Engineers, Erie, PA, USA) equipped with a vacuum pump, condenser, and cold trap for collecting the by-products. In the first step, the reactor was charged with DMFDC and HDO, with the molar ratio of the diester (DMFDC) and glycol (HDO) of 1:1.5, and catalyst. The transesterification reaction was carried out under a constant flow of nitrogen at the temperature of 160–180 °C for ca. two hours. During this stage, one distilled and collected the first by-product, methanol. The progress of the transesterification reaction was monitoring based on the amount of effluent by-product. When the distillation of methanol has ceased (ca. 90% of the stoichiometric amount), the reaction was completed, and we gradually increased the temperature up to 210 °C. Subsequently, polyTHF^®^1000, thermal stabilizer, and the second portion catalyst were introduced to the reactor. The reaction temperature was increased to 240 °C (for neat polyester, PHF) and 235 °C (for the series of block copolymers). The vacuum was applied gradually in order to facilitate HDO excess removal and the final pressure was lower than 25 Pa. The stirring torque change was monitored to evaluate the melt viscosity of the product, and thus the progress of this step of the synthesis. The polycondensation process was found to be finished when the reaction mixture reached the appropriate value of melt viscosity (referring to a high molecular mass of the polymer material). Finally, the material was extruded from the reactor into the water bath using compressed nitrogen. The second stage reaction time was 4–6 h, dependent on the weight content of pTHF segments. The PHF-*b*-F-pTHF copolymers with several weight ratios of rigid (PHF) to flexible (F-pTHF) segments (75/25, 65/35, 50/50, 35/65, 25/75) were prepared. The homopolymer PHF was synthesized following the same procedure (without the addition of pTHF in the second step).

All of the obtained materials were pelletized and injection moulded using Boy 15 (Dr BOY GmbH&Co., Neustadt, Germany) to obtain dumbbell shape samples, type A3, for DMTA and tensile measurements. Samples before the injection molding were dried for 24 h under a vacuum at the temperature of 50 °C. The following parameters were used: injection pressure 50 MPa; melt temperature: 15 °C higher than the melting point of the polymer determined by DSC; mold temperature 30 °C, holding down pressure of 20 MPa for 15 s and cooling time of 10 s.

### 2.2. Characterization Methods

Attenuated total reflectance—Fourier Transform Infrared (ATR-FTIR) spectra of PHF and PHF-*b*-F-pTHF copolymers were recorded using FTIR spectrophotometer Tensor 27 (Bruker OptikGmbH) with 32 scans and a resolution of 2 cm^−1^ over the frequency range of 4000–400 cm^−1^.

The molecular structure and composition of the prepared materials were determined using ^1^H NMR spectroscopy. Before the experiment, to remove unreacted monomer and any possible low molecular degradation products, all samples were subjected to continuous Soxhlet extraction with methanol. ^1^H NMR spectra were recorded at room temperature with a Bruker spectrometer operated at 400 MHz. The samples were dissolved in chloroform-d CDCl_3_ at a concentration of 10 mg/mL. Tetramethylsilane (TMS) was used as an internal chemical shift reference.

The number average ($\bar{M_{n}}$)) and weight average molecular masses $(\bar{M_{w}}$), as well as polydispersity index (M_w_/M_n_), were evaluated using Size Exclusion Chromatography (SEC) in 1,1,1,3,3,3-hexafluoroisopropanol (HFIP) at 40 °C, on a system equipped with a Waters 1515 Isocratic HPLC pump, a Waters 2414 refractive index detector (35 °C), a Waters 2707 autosampler, and a PSS PFG guard column followed by two PFG-linear-XL (7 μm, 8 × 300 mm) columns in series. HFIP with potassium trifluoroacetate (3 g/L) was used as the eluent at a flow rate of 0.8 mL/min. Calibration of the system was performed concerning poly(methyl methacrylate) standards.

The intrinsic viscosity [IV] of the series of copolymers was determined at 30 °C in the mixture of phenol/1,1,2,2-tetrachloroethane (60/40 by weight). The concentration of the polymer solution was 0.5 g/dL. The measurement was carried using a capillary Ubbelohde viscometer (type Ic, K = 0.03294).

The phase structure of PHF and PHF-*b*-F-pTHF copolymers was investigated using differential scanning calorimetry (DSC), dynamic mechanical thermal analysis (DMTA), and X-ray diffraction (XRD). DSC analysis was performed using Mettler Toledo differential scanning calorimeter (DSC1, was calibrated for the temperature and melting enthalpy by using indium and n-octane as a standard) under nitrogen atmosphere in the heating-cooling-heating cycle, with the heating/cooling rate of 10 °C/min, from −120 to 225 °C. The characteristic phase transition temperatures (glass transition and melting) were taken from the second heating run. Glass transition temperature (T_g_) was determined using the midpoint approach, while specific heat capacity (ΔCp) was calculated from the vertical distance between extrapolated baselines at T_g_. The crystallization and melting temperatures (T_c_ and T_m_, respectively) were determined from the maximum of the exothermic and endothermic peaks, respectively. The heat of fusion (ΔHm) and crystallization (ΔHc) were calculated from the total areas under melting and crystallization peaks on the DSC curve. The softening temperatures were also determined with Boetius apparatus (HMK 71/3407, Franz Küstner Nachf. KG, Dresden, Germany), where one observes the changes in polymers structure along with an increase in temperature, and thus observing the moment (temperature range), in which the crystalline phase will completely disappear. The DMTA analysis was performed using a DMA 242 E/1/G Artemis (Netzsch, Selb, Germany) apparatus working in a bending mode in a temperature range from −100 °C to the polymer melt temperature, at a frequency of 1 Hz and a heating rate of 3 °C/min. The properties were determined based on modulus changes and the ability of attenuation as a function of temperature and frequency of load changes. The XRD analysis of the materials was conducted with the use of a Panalytical X’Pert diffractometer operating at 40 V and 40 mA with CuKα radiation (λ = 0.154 nm). The samples were scanned from 2θ = 4° to 60° with a step of 0.05°.

The morphology of the nanocomposites was analyzed using a scanning electron microscope (FE-SEM, Hitachi SU-70). Before SEM analysis, the samples were cryofractured in liquid nitrogen and then coated with a thin film of palladium-gold alloy, using a thermal evaporation PVD (physical vapor deposition) method.

The shape memory performance was monitored using cyclic thermo-mechanical analysis using a dynamic mechanical analyzer (DMA Q800, TA Instruments) working in a controlled strain mode. Polymer films of approximately 200 μm thick were tested. The measurements were performed following the procedure described in detail elsewhere [39,40]. Seven consecutive cycles consisting of heating—stress loading—cooling—stress unloading—heating were conducted. Programing of SMP was carried out at 20 °C, while fixing of temporary shape was performed at −50 °C. A constant heating and cooling rate of 10 °C/min were maintained. The shape fixity efficiency (R_f_) and shape recovery (R_r_) were determined according to the following equations:

(1) $$R_{\mathrm{Nf}}=\frac{\varepsilon_{N2}-\varepsilon_{N0}}{\varepsilon_{N1}-\varepsilon_{N0}}\times 100\%$$

(2) $$R_{\mathrm{Nr}}=\frac{\varepsilon_{N2}-\varepsilon_{N0}}{\varepsilon_{N2}-\varepsilon_{N0}}\times 100\%$$

where: ε_N0_ = the initial strain for the Nth cycle, ε_N1_ = the maximum strain of stretched sample, ε_N2_ = the fixed strain after unloading, ε_N0_ = the strain after recovering.

The static mechanical properties were measured using an Autograph AG-X plus universal testing machine (Shimadzu, Duisburg, Germany) equipped with a 1 kN Shimadzu load cell, a noncontact optical extensometer, and the TRAPEZIUM X computer software operated at a constant crosshead speed of 100 mm/min. Measurements were performed at room temperature on the dumbbell samples with a grip distance of 20 mm. According to PN-EN ISO 527 standard, Young’s modulus, tensile strength (σ_y_) and elongation at yield (ε_y_), strength at break (σ_b_) and elongation at break (ε_b_) of PHF and block copolymers were determined. The reported values are the mean values of ten measurements. Cyclic tensile measurements were performed only for PHF-*b*-F-pTHF copolymers using the same equipment and a crosshead speed of 100 mm/min. Samples were stretched until the specified strain value was reached, and then the tensile force was released to zero. This procedure was repeated, with increasing deformation value, until the sample broke. The following strains were established for our test: 5%, 15%, 25% 50% 100%, 200%, and 400%. Moreover, the Shore D hardness was measured using a Zwick 3100 Shore D tester (Zwick GmbH, Ulm, Germany). Each reported value was the mean of 20 independent measurements.

The thermo-oxidative and thermal stability of the synthesized homopolymer PHF and PHF-*b*-F-pTHF copolymers were evaluated by thermogravimetry (TGA 92-16.18 Setaram, Caluire-et-Cuire, France). Measurements were carried out in an oxidizing atmosphere, i.e., dry, synthetic air (N_2_:O_2_ = 80:20 vol.%) and in an inert atmosphere (argon) at a flow rate of 20 mL/min. The study was conducted at a heating rate of 10 °C/min in the temperature range of 20–700 °C. Measurements were conducted in accordance with the PN-EN ISO 11358:2004 standard.

## 3. Results and Discussion

### 3.1. Analysis of Structure and Composition

**Figure 1:**
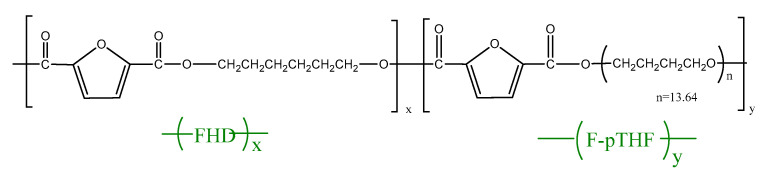
Structure of poly(hexamethylene 2,5-furanodicarboxylate)-block-poly(tetrahydrofuran) (PHF-*b*-F-pTHF copolymers).

A series of thermoplastic poly(ether-ester)s based on renewable raw materials, where a hard phase was most often the crystallites of PHF located in a soft amorphous matrix (soft phase) consisting of polyether sequences (F-pTHF), was synthesized by a catalyzed two-stages reaction, involving transesterification, and polycondensation in the bulk. All materials were synthesized using a highly effective catalyst, Ti(OBu)_4_), and antioxidant (Irganox 1010), which were typically used by our group to obtain block copolymers [41,42,43,44,45]. The PHF-*b*-F-pTHF copolymers can be considered as random copoly(ether-esters) (F units and F-pTHF units), and their molecular formula is shown in Figure 1. One can calculate the degree of polycondensation of the rigid segment (F)x. In addition, the theoretical chemical composition and the chemical composition estimated from 1H NMR analysis, along with their basic physic-chemical properties, utilizing intrinsic viscosity [η], number (M_n_) and weight (M_w_) average molecular masses, and polydispersity index (PDI) are summarized in Table 1.

The degree of polymerization (x) of the rigid PHF segment was calculated according to 1 mol (y = 1 in Figure 1) of flexible F-pTHF (where F states for furanoate unit) segments. The value of x of the rigid segments (Table 1), calculated from the composition of the reaction mixture, is ranging from 14.2 (PHF-*b*-F-pTHF 75/25) to 1.57 (PHF-*b*-F-pTHF 25/75).

The limiting viscosity number [η] of polymers/copolymers can be influenced by the molecular chains’ weight and their flexibility [41]. Moreover, it was found for PHF that also the condensation temperature can affect the intrinsic viscosity [32]. In the present study, the value of [η] for PHF homopolymer equals 0.532, which was slightly lower than that for the previously published in [32], with comparable parameters of the polycondensation process (temperature of 230 °C). In contrast, the values of intrinsic viscosity for the series of copolymers increased along with an increase of weight fraction of F-pTHF segments from 0.696 dL/g to 0.922 dL/g (Table 1). All copolyesters exhibited high number and weight average molecular masses, ca. 27,000 (M_n_) and ca. 70,000 (M_w_), respectively. Only neat PHF was characterized by lower masses (M_n_ slightly below 20,000, and M_w_ about 54,000), which resulted from the fact that all materials were attempted to be obtained under similar conditions for comparison purposes (polycondensation was carried out in time, with the time of this stage being approx. 3h). Therefore, under comparable synthesis conditions to copoly(ether-esters), it was possible to obtain a material with a slightly lower molecular weight than in the literature [32]. In turn, the values of PDI for all synthesized materials were above 2 (Table 1). These values were similar for all polymers and copolymers synthesized using the polycondensation procedure [40,45,46]. This phenomenon can be explained by the fact that when two different monomers are involved (with different molecular masses), there is a stoichiometric imbalance and the classical Flory’s most probable distribution might be no longer valid since the equation for a rigorous calculation of the PDI becomes very complex.

**Figure 2:**
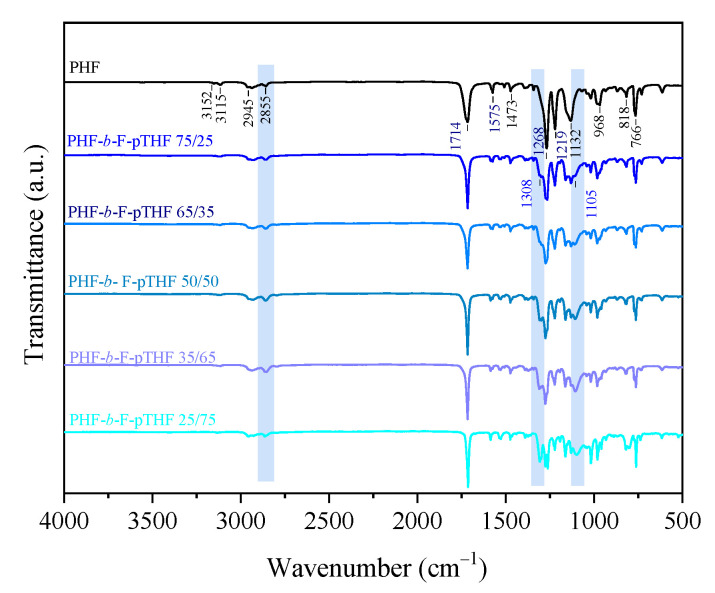
Fourier Transform Infrared (FTIR) spectra of PHF homopolymer and PHF-*b*-F-pTHF copolymers.

The PHF and copolymers structure was investigated by means of FTIR spectroscopy. Figure 2 presents the FTIR spectra of neat PHF and PHF-*b*-F-pTHF copolymers, in which strong absorption peaks originating from the C=O and C(=O)-O stretching mode of ester groups occur at 1714 cm^−1^ and 1268 cm^−1^, respectively. One can also observe absorption peaks corresponding to 2,5-disubstituted furan heterocycles at ~3126 cm^−1^ and ~3162 cm^−1^ (=C-H stretching vibrations in the furan ring), ~1580 cm^−1^ (aromatic C=C stretching vibrations), ~1218 cm^−1^ (=C-O-C= ring vibrations). Moreover, some weak signals ascribed to furan ring out-of-plane deformation appeared at ~960 cm^−1^, ~818 cm^−1^, and ~766 cm^−1^ [29,46,47,48,49]. Close inspection of PHF-*b*-F-pTHF copoly(ether-esters) spectra in highlighted regions reveal the evolution of additional peaks within the incorporation of polyether soft segment. In particular, additional absorption peaks due to C-O-C stretching vibration of pTHF appear at a wavelength of 1308 cm^−1^ and 1105 cm^v1^. Similar phenomenon were observed by Chi and co-workers who copolymerized poly(neopentyl glycol 2,5-furandicarboxylate) (PNF) with poly(tetramethylene glycol) (PTMG) [49]. As expected, along with an increase of pTHF co-unit content, a gradual increase in the intensity of absorption band at 2855 cm^−1^ can be observed due to -C-H asymmetric and symmetric stretching vibrations in -CH_2_- groups. The lack of the absorption band at ~3450 cm^−1^ characteristics for stretching of -OH group suggests complete consumption of terminal pTHF hydroxyl group through copolymerization; thus, confirming the success of modification.

**Figure 3:**
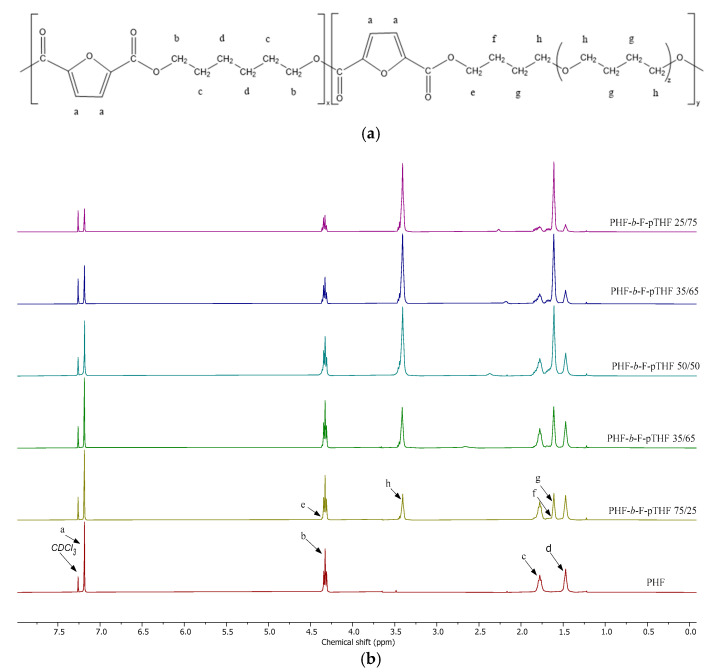
Structure of PHF-*b*-F-pTHF copolymers (**a**) and nuclear magnetic resonance (NMR) spectra of PHF-*b-*F-pTHF copolymers (**b**).

The chemical structure of PHF-*b*-F-pTHF poly(ether-ester)s was further assessed by quantitative ^1^H NMR studies. The chemical shifts and corresponding peak assignments are reported in Figure 3. In the ^1^H NMR spectra of homopolymer, the resonance corresponding to the two furanoate ring protons appear as a singlet signal at 7.09 ppm (a signal), whilst those corresponding to the outer (b signals), inner (c signals), and middle (d signals) methylene protons of hexylene glycol units occur at 4.23 ppm, 1.63 ppm, and 1.31 ppm, respectively. These peak assignments agree well with the data reported earlier by Jiang et al. [29] and Papageorgiou et al. [21]. All of the resonances mentioned above can also be distinguished in the copolymer spectra, with their intensity being dependent on the ratio of FHD to F-pTHF segments. Besides, a number of new resonance signals attributable to the protons of pTHF blocks occur at copolymers spectra. New peaks occurring at 1.60 ppm (h signal) and 3.40 ppm (g signal) can be reasonably ascribed to outer and inner methylene protons of pTHF ether blocks, respectively [50]. Furthermore, new less intense signals occurred in the methylene group proton region owing to pTHF protons located in a different environment (adjacent to PHF segment by ester bond) after transesterification reaction between the various units, i.e., PHF and pTHF. The methylene proton resonances of pTHF segments attached to PHF can be observed at 4.36 ppm (e signal, partially overlapped with b signal) and at 1.64–1.71 ppm (f signal). It is clear that the intensity of both pTHF main chain peaks (g and h signals) and cross-peaks (e and f signals) depends on the content of ether co-units and increases sharply as the ratio of pTHF increases.

The relative area ratio of the ^1^H NMR resonance peaks attributed to the aromatic protons of the furan ring at δ~7.15–7.22 ppm and inner methylene protons of the p-THF at δ ~1.53–1.65 ppm were employed to estimate the actual weight content of F-pTHF segments following Equation (3):

(3) $$W_{\mathrm{FpTHF}}\left(\mathrm{wt}\%\right)=\;\frac{\left(\frac{I_{g}}{6}\right)\times 210}{\left(\frac{I_{g}}{6}\right)\times 210+\;\left(\frac{I_{a}}{2}\right)\times 238.24}\times 100\%$$

where I*_g_* and I*_a_* are the internal signal intensities for the corresponding peak at δ ~1.53–1.65 (g) and δ ~7.15–7.22 ppm (a), respectively; 210 and 238.24 are the molecular weights of the repeating units of F-pTHF and PHF segments, respectively.

The calculated copolymer composition (Table 1) was found to differ from the theoretical values by less than a few percent. These slight differences were already expected and can be related to (i) the introduction of a certain excess of p-THF added to theoretical calculations due to the losses in the dosing of the viscous substrate (marked especially at low p-THF concentrations), and (ii) distillation of the reaction substrate under reduced pressure. A similar tendency was also reported by Chi et al. [49] for PNF-PTMG copolymers, and the authors attributed this effect to the sublimation of NPG during polycondensation. Nonetheless, the obtained results confirm that the copolymers’ composition can be controlled simply by varying the substrate feed ratio of DMFDC and HDO to pTHF.

### 3.2. Phase Structure and Morphology

**Figure 4:**
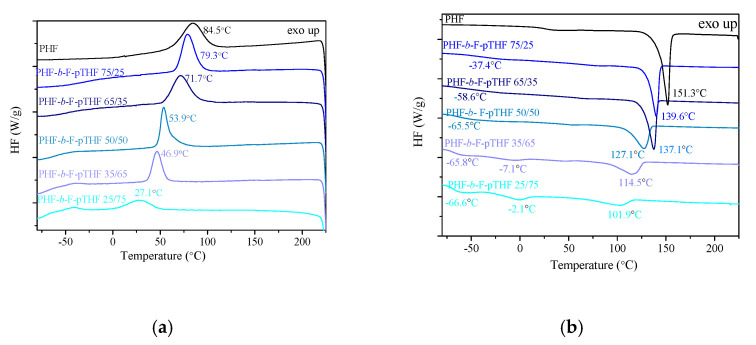
Differential scanning calorimetry (DSC) thermograms for homopolymer PHF and PHF-*b*-F-pTHF copolymers recorded during: (**a**) cooling and (**b**) second heating.

In PHF-*b*-F-pTHF copolymers, similarly to other segmented copolymers, the PHF and F-pTHF segments can separate into discrete phases when solidified from the melt results from the thermodynamic immiscibility of the rigid (F) and flexible (F-pTHF) segments. The occurrence of this hetero-phase structure in the studied copolymers, resulting from semicrystalline and amorphous domains, was studied and confirmed by DSC and DMTA analyses. The DSC thermograms recorded during cooling and second heating are presented in Figure 4. The glass transition temperature of PHF can be found to be T_g_ = 16.7 °C (with corresponding ΔC_p_ of 0.26 J/g), which was slightly higher than the values obtained by Papageorgiou et al. [21], but their experiment was conducted using a heating rate of 20 °C/min (after cooling at 80 °C/min). We also confirmed that PHF was a semicrystalline sample, that crystallized during cooling with the crystallization temperature of ca. 84 °C. The melting temperature of PHF was found to be T_m_ = 151.3 °C. For block copolymers, along with the increasing content of pTHF, we observed the decrease in the phase transition temperatures (T_m_ and T_c_) and the corresponding enthalpies, melting (ΔH_m_) and crystallization (ΔH_c_), respectively. When comparing the sample with the highest content of F-pTHF segments (75 wt.%) to the lowest content (25 wt.%), these differences amount to over 40 °C for the melting temperatures and almost 60 °C for the crystallization temperatures, due to the decreasing content of the semicrystalline PHF-rich phase. For all materials, only one T_g_ is observed, and the value of which depends on the composition of the copolymer. In addition, on the DSC curves of PHF-*b*-F-pTHF 35/65 and PHF-*b*-F-pTHF 25/75, i.e., with the highest content of pTHF, one can observe the melting of crystallites of pTHF at the temperatures −7.1 °C and −2.1 °C, respectively. One can find pTHF as a semicrystalline polyether [51], and in block copolymers with long pTHF sequences, the crystallites of pTHF can be observed [52].

**Figure 5:**
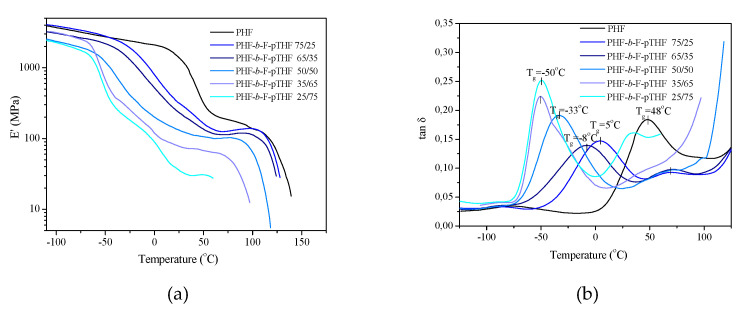
The storage modulus E’ (**a**) and tan δ (**b**) as a function of temperature for PHF-*b*-F-pTHF copolymers.

The dynamic mechanical properties of PHF and PHF-*b*-F-pTHF copolymers as a function of temperature are shown in Figure 5. The values of storage modulus at 25 °C are also summarized in Table 2. The properties of the copolymers change with the temperature change in a manner characteristic for thermoplastic elastomers, i.e., in the curve of the storage modulus (E’), there is an inflection related to the soft phase glass transition, defining the lower temperature of the elasticity interval. The range of the flat course of these curves (the so-called “wide plateau of elasticity”) determines the possibility of using the obtained copolymers (especially with a higher content of pTHF as thermoplastic elastomers. The distinct differences between the PHF and the copolymers are explicitly visible in the values of storage modulus determined at 25 °C (Table 2), from 1373 MPa (homopolymer PHF) to 35.2 (for PHF-*b*-F-pTHF 25/75). From the curves of tan δ (Figure 5b), one can observe that the PHF homopolymer has the α-relaxation at 48 °C corresponding to the glass transition of the amorphous part in the semicrystalline hard phase. One beta relaxation associated with amorphous soft phase transition shifted towards higher temperatures, as the result of the increasing presence of non-crystallized polyester PHF sequences in this phase is visible. The amorphous soft phase is a blend of polyether (pTHF) sequences and non-crystallized polyester (PHF) sequences, which can restrict thermal motions within pTHF-rich soft segment regions. Herein, also along with the increasing content of pTHF, the maxima of the peaks are shifted toward lower values. Two samples with the highest amount of F-pTHF segments (65 and 75 wt.%) exhibited similar values of −50 °C.

Additionally, for the synthesized materials, the Boetius softening temperature range was determined, and the values are summarized in Table 2. This measurement is particularly useful for determining material processing temperatures when obtaining samples employing the injection molding process. For this measurement, the temperature range was given at which the sample began to soften when observed under a microscope. The influence of the chemical composition was visible, and, as the soft phase content increased, the softening temperature range was shifted towards lower values. This confirmed the observations from DSC analysis concerning the decrease of T_m_ as a function of copolymer composition.

**Figure 6:**
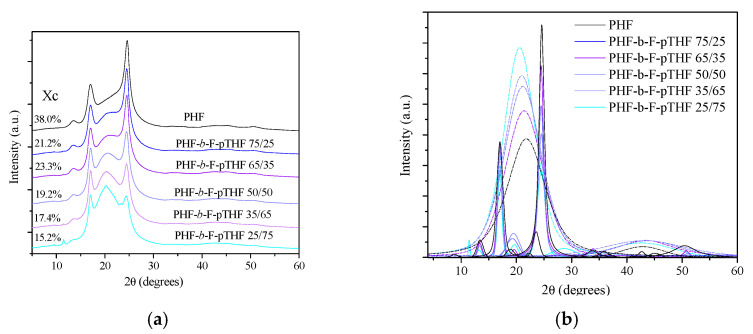
X-ray diffraction (XRD) curves of PHF-*b*-F-pTHF copolymers (**a**) with separated crystalline peak positions and amorphous halo reflections (**b**).

To investigate the crystal structure of the obtained copolymers, XRD analysis was performed. Figure 6 presents WAXS patterns of neat PHF homopolymer and PHF-*b*-F-pTHF copolymers. The obtained PHF had typical (110) plane, (010) plane, and (111) plane at 2θ = 13.31° (d = 6.64 Å), 17.06° (d = 5.19 Å), and 24.46°(d = 3.63 Å) [32]. Unlike other polyesters based on 2,5-furanedicarboxylic acid, such as poly(ethylene 2,5-furanoate) (PEF) or (poly(butylene 2,5-furanoate) (PBF), PHF is highly crystalline at room temperature, with the degree of crystallinity greater than 35% [53]. As the content of the flexible F-pTHF segment increases, the degree of crystallinity of the sample decreases (Figure 6a). The same trend was observed in the DSC analysis. A decreasing degree of crystallinity with an increase in the content of the flexible segment and unchanging positions of the crystalline peaks may indicate that only the PHF crystal structure exists in the obtained copolymers. The main reflections at 2θ = 17.06° and 24.46°, of copolymers with predominant F-pTHF segments, lose their sharp shape, which also indicates the amorphous character of F-pTHF.

**Figure 7:**
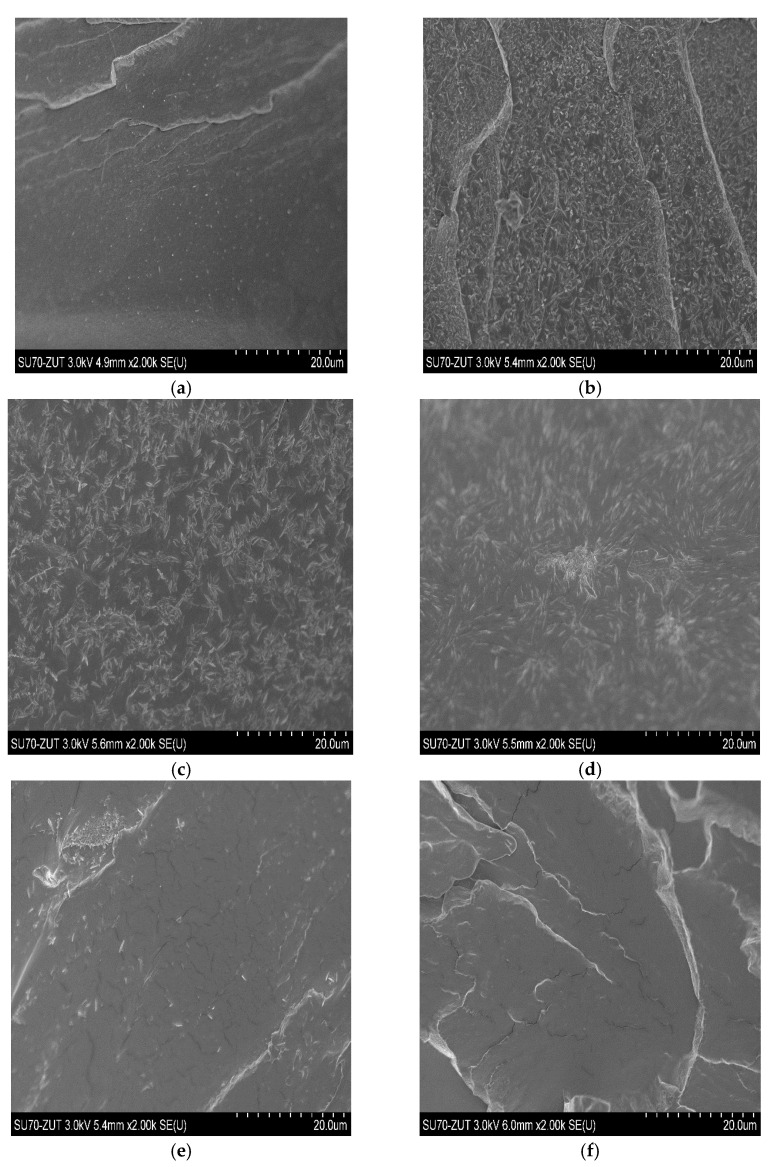
Scanning electron microscopy (SEM) micrographs of PHF, (**a**) and PHF-*b*-F-pTHF copolymers, 75/25 (**b**), 65/35 (**c**), 50/50 (**d**), 35/65 (**e**), and 25/75 (**f**).

Observations carried out by scanning electron microscopy (SEM), significant from the point of view of the material structure analysis, confirmed the heterogeneity of the structure of the PHF-*b*-F-pTHF copolymers tested. Although both phases are immiscible, they exert a significant influence on each other. The micrographs of the fractures of the samples are presented in Figure 7. The obtained images showed differences in the phase structure depending on the composition of the copolymers. Figure 7a shows the spherulitic morphologies of the PHF. With the increase in the proportion of pTHF in the copolymer, spherulites became larger, but their number decreased (Figure 7b–f), which was confirmed by the DSC research. Based on the shape and temperature position of the crystallization peaks on the DSC curves determined during the cooling of the molten polymer with a constant speed (Figure 4a), it can be seen that the crystallization rate influenced the crystallite morphology. The crystallization peaks of PHF-*b*-F-pTHF 75/25 and PHF-*b*-F-pTHF 65/35 samples were characterized by a wide base (wide temperature range), which means that at this cooling rate, crystallization took place more slowly than for others. This was manifested by more crystalline forms with a more regular shape. Faster crystallization favors irregular forms.

The observations and analysis of the mechanical tests also showed that the tensile strength of the obtained polymers depended mainly on the content of the crystallization segment and decreased with its loss. On the other hand, the elongation at break depended on the amorphous segment, and as its content increased, it also increased.

### 3.3. Shape Memory Behavior

**Figure 8:**
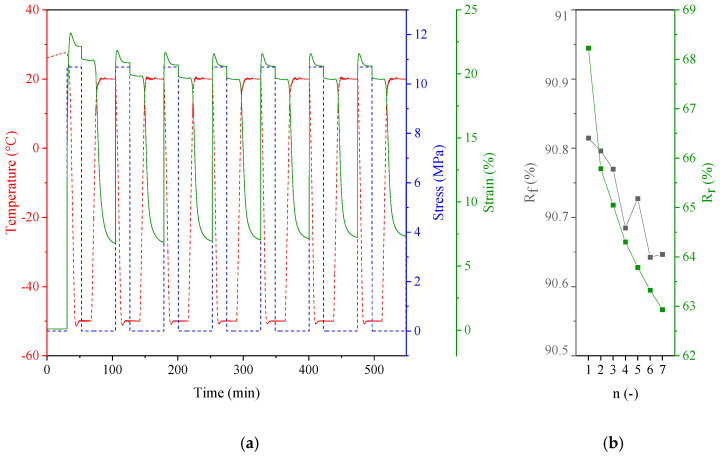
Shape memory properties of PHF-*b*-F-pTHF 25/75: (**a**) multiple thermomechanical cycles and (**b**) shape fixity (R_f_) and shape recovery efficiency (R_r_) vs. cycle number.

As previously demonstrated [40], copolymers characterized by glass transition temperature accompanied with a sudden gain in molecular mobility (i.e., a high value of ∆Cp) and sufficient elastic ratio of glassy to rubbery modulus (E’_g_/E’_r_) may behave as a shape memory polymer (SMP). In addition to the glass transition temperature, melting temperature (T_m_) or liquid crystalline (LC) phase transition have been reported as stimuli-sensitive switches [54,55,56]. Based on DSC and DMTA results described in the previous section, one can deduce that PHF-*b*-F-pTHF copolymers containing the highest amount of soft segments (75 wt.%) can be qualified for further studies on shape memory effect, induced by an external stimulus, i.e., temperature (T_trans_). In principle, T_trans_ can be either glass transition temperature (T_g PHF-*b*-F-pTHF 25/75_ = −66.6 °C) or low temperature melting (T_m PHF-*b*-F-pTHF 25/75_= −2.8 °C). The latter was considered as a switching transition temperature in the present work. More specifically, a cyclic thermomechanical analysis was employed to evaluate the SMP ability to fix mechanical deformation and recover to its original shape. Each cycle consisted of hating to 20 °C (calorimetric T_m_ + 20 °C), stretching the film sample by ramping a load of 10 N, cooling at constant load to a “fixing temperature,” which was well below melting of PHF-*b*-F-pTHF 75/25 (−50 °C) and finally reheating back to 20 °C. After unloading, the sample contracts slightly and retains its new, temporary shape. When heated above T_trans_, the second, more pronounced contraction occurs. The extend of this return quantifies the ability of SMP to recover the original shape. The consecutive thermomechanical cycles are reported in Figure 8a, whilst the calculated values of the shape fixity efficiency (R_f_) and shape recovery (R_r_) were plotted as a function of cycle number in Figure 8b. From the obtained results, it is apparent that the investigated copoly(ether-ester)s exhibited a relatively high ability to fix mechanical deformation since R_f_ remained high and almost independent of the cycle number (90.6 ≤ R_f_ 90.8%). On the other hand, R_r_ varied slightly in the individual cycles. The highest R_r_ difference could be observed between the first and successive thermomechanical cycles. This phenomenon occurred in most thermoplastic SMPs [39,57] and seemed to be related to sample processing history. Anyway, based on the conducted studies, one can state that PHF-*b*-F-pTHF 25/75copolymer can be applied as a promising SMP polymer, with a shape fixity of over 90% and shape recovery efficiency of over 60%. The mechanism of its shape memory behavior was triggered by the melting of switching segment crystals (provided by the pTHF crystalline phase).

### 3.4. Mechanical and Elastic Properties of PHF-b-F-pTHF

**Figure 9:**
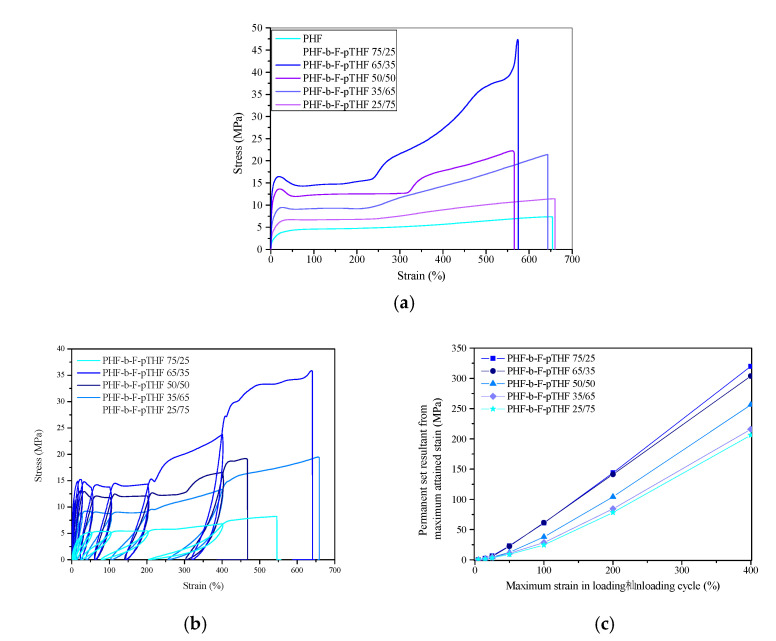
Representative stress–strain curves for PHF-*b*-F-pTHF copolymers from (**a**) static tensile tests, (**b**) cyclic tensile tests, and (**c**) permanent set results after following deformation cycle.

The representative stress-strain curves for PHF and PHF-*b*-F-pTHF copolymers are presented in Figure 9. Moreover, the values of Shore hardness, tensile modulus (E), tensile strength at yield (σ_y_) elongation at yield (ε_y_), stress at break (σ_b_), and strain at break (ε_b_) are summarized in Table 3. The obtained PHF is characterized by high stiffness and low strain at break, which differed from the results reported in the literature [38,53]. The differences may result from the greater degree of crystallinity of the material, but also from the higher stretching speed, which in our case was 100 mm/min. Due to the high strain rate, the PHF might not have enough time to adjust to the deformation, which may result in a low value of elongation at break. With the increase in the proportion of the flexible pTHF segments in the copolymer, the Young’s modulus and the tensile strength, as well as hardness decrease. The copolymer with the highest content of flexible segments was characterized by a hardness lower by over 60% and a Young’s modulus over 35 times lower than the PHF homopolymer. The obtained results were consistent with the expectations. F-pTHF segments are more elastic, and with their higher content, the degree of crystallinity of the samples decreases. On the other hand, the values of elongation at break significantly increase already at 25 wt.% of the pTHF segments, achieving the highest value of 665% for PHF-*b*-F-pTHF 35/65 copolymer. Copolymers with a dominance of more rigid polyester segments were characterized by a clear yield point. Moreover, a strain hardening effect caused by macromolecular chains orientation and crystallization during stretching was observed. Since copolymers with a greater content of pTHF segments are characterized by a lower crystallization ability, the strengthening effect in their case is greatly reduced.

Figure 9b shows the representative stress-strain curves for PHF-*b*-F-pTHF copolymers under cyclic tensile loading. The main contours of the curves obtained in the cyclic tensile tests, reflect the trend observed under uniaxial static tensile tests. To assess the elastic recovery of the obtained copolymers, Figure 9c shows the values of a permanent set level after each deformation cycle. As the strain increases, the differences in the values of the residual strain of materials are more and more pronounced. As exhibited, copolymers with a predominance of flexible pTHF segments have better recovery properties. The higher stiffness of the PHF segments limits the material return after deformation. Besides, the higher the degree of crystallinity of the material, the more difficult it is to restore the original shape and the permanent set level is higher. Therefore, as expected, it can be concluded that copolymer PHF-*b*-F-pTHF 25/75 has the best elastic properties (the lowest values of the permanent set).

### 3.5. Thermo-Oxidative and Thermal Stability

**Figure 10:**
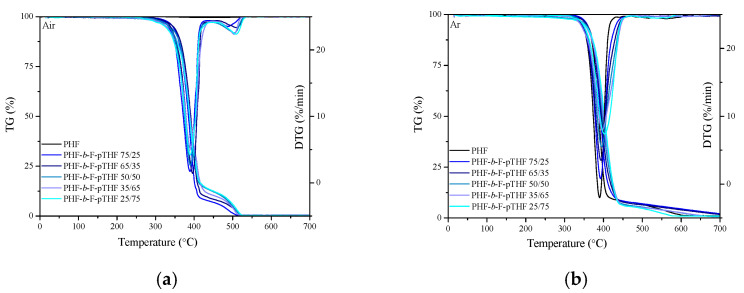
Mass loss (TG) and derivative of mass loss (DTG) curves for PHF-b-F-pTHF copolymers measured in an oxidizing atmosphere (air) (**a**) and in an inert atmosphere (argon) (**b**) at the heating rate 10 °C/min.

The applicative performance of polymer materials depends largely on their stability at higher temperatures. The thermo-oxidative and thermal stability of the synthesized PHF-*b*-F-pTHF copolymers and PHF homopolymer were analyzed using thermogravimetry under oxidative (air) atmosphere (Figure 10a) and argon (Figure 10b). The characteristic temperatures for the mass of 5%, 10%, and 50% (T_5%_, T_10%_, T_50%_) argon and air, along with the temperatures corresponding to the maximum of mass losses (T_DTG1_ and T), activation energies (E_a_) and correlation coefficient in linear regression (R) are summarized in Table 4. One can consider the value of T_5%_ as the beginning of thermal degradation. It can be seen that along with the amount of pTHF the decrease in the value of T_5%_ was observed. In turn, at higher temperatures, no such significant differences in thermo-oxidative stability as a function of copolymer composition were observed, especially in the case of T_50%_ (differences in the size of 1–2 °C). Likewise, the values of T_DTG1_ and T_DTG2_ were comparable to each other and practically independent from the composition. The whole series of materials in an oxidizing atmosphere exhibited two stages of degradation, which appeared at 270–445 and 455–540 °C (Figure 10a), respectively. The first stage is attributed to the decomposition of flexible and rigid segments. We know that the oxygen attack on poly(ether-ester) block copolymers is initiated in the flexible segment and, in most cases occurs at the α-carbon atom to the ether oxygen atom [42,58] and results in the formation of volatiles, while the second stage is attributed to the decomposition of the residue. Besides, from the shape of the TG and DTG curves, it can be observed that in an inert atmosphere (argon) the thermal behaviors of PHF-*b*-F-pTHF copolymers and PHF homopolymer are very similar and practically independent from the composition. Nevertheless, the values of activation energy (E_a_) was strongly dependent on the composition, in both oxidizing and inert atmosphere. The values of E_a_ for PHF homopolymer and PHF-*b*-F-pTHF 75/25 were comparable, but along with the increasing amount of pTHF, a significant decrease was visible, from about 320 kJ/mol to about 249 kJ/mol, if compared samples with the highest (75 wt.%) and lowest (25 wt.%) of pTHF, in an oxidizing atmosphere (Table 4). Similar behavior was observed in an inert atmosphere (decrease of ca. 140 kJ/mol). The weakest bond in the PHF-*b*-F-pTHF copolymer backbone and, therefore, the most susceptible to scission at lower temperatures is the C–O bond occurring in pTHF segments. Therefore, it has been observed that with increasing content of PTHF in copolymers, the energy of activation for copolymer decomposition is decreasing and thus the stability [58].

## 4. Conclusions

The series of bio-based block copolymers, by means of PHF-*b*-F-pTHF were successfully synthesized using a two-stage polymerization method that consists of transesterification and polycondensation in the melt. It was proven that the employment of renewable raw materials (dimethyl 2,5-furanodicarboxylate, Henan Coreychem Co., China; 1,6-hexylene glycol, Rennovia Inc., USA, and pTHF, BASF) allowed obtaining polyester (PHF) and poly(ether-esters) (PHF-*b*-F-pTHF) with high molecular masses and intrinsic viscosities, thus opening a relatively broad range of future applications that may compete with petroleum-based thermoplastic polyesters and copolyesters. Of course, nowadays, it is still economically not profitable to completely replace TPA by FDCA, or fossil-based PTMO with bio-based pTHF, due to relatively high market prices of the monomers, however, falling prices (from year to year) and increased public awareness are contributing to more and more prospective applications for this types of materials. Therefore, it is of great importance to synthesize (especially in “macro-laboratory” scale, or even “half-industrial” scale) polyesters and copolyesters based on renewable raw materials, and characterize them in detail, which will significantly contribute to a faster transfer of the production of this type of materials to an industrial scale. Therefore, in the present study special emphasis was placed on the accurate structure-property relationship. The PHF homopolymer and PHF-*b*-F-pTHF copolymers structures were investigated utilizing FTIR spectroscopy and ^1^H quantitative nuclear magnetic resonance (NMR), and it was found that the real composition was found to be very close to the one theoretically calculated. DSC and DMTA analyses, along with SEM observations, provided information on the phase structure of PHF-*b*-F-pTHF copolymers. In the obtained copolymers, the hetero-phase structure resulting from semicrystalline and amorphous domains was visible. Moreover, it was found that PHF-*b*-F-pTHF 25/75 copolymer can be applied as a promising SMP polymer, with a shape fixity of over 90% and shape recovery efficiency of over 60%. Besides, the static and cyclic tensile performance of PHF-*b*-F-pTHF copolymers confirmed their ability to relatively high deformations (especially with the highest content of pTHF, i.e., 75 wt.%) and damping ability at room temperature. In addition, it was proved that the synthesized copolymers can be processed by injection molding and exhibited high thermo-oxidative and thermal stability, where the increasing content of the soft phase did not contribute to a significant decrease in the stability, which prevents their degradation during processing (at higher temperatures).

## Figures and Tables

**Table 1:** Composition and molecular characterization of poly(hexamethylene 2,5-furanodicarboxylate)-block-poly(tetrahydrofuran) (PHF-*b*-F-pTHF) copolymers.

Sample	x [mol]	w_PHF_ [wt.%]	w_FpTHF_ [wt.%]	W_FpTHF_^NMR^ [wt.%]	[η] [dl/g]	M_n_ [g/mol]	M_w_ [g/mol]	PDI
PHF	-	100	-	-	0.532	19,200	54,100	2.72
PHF-*b*-F-pTHF 75/25	14.12	75	25	31.98	0.696	27,500	71,000	2.58
PHF-*b*-F-pTHF 65/35	8.73	65	35	40.95	0.837	26,400	69,200	2.62
PHF-*b*-F-pTHF 50/50	4.71	50	50	58.51	0.865	27,500	70,900	2.58
PHF-*b*-F-pTHF 35/65	2.53	35	65	64.60	0.893	26,400	68,800	2.61
PHF-*b*-F-pTHF 25/75	1.57	25	75	72.18	0.922	26,900	74,700	2.77

x, degree of polymerization of F segment; w_PHF_, weight content of PHF segments; w_FpPHF_, weight content of F-pTHF segments in block copolymer calculated theoretically and from NMR spectra; [η], intrinsic viscosity; M_n_, M_w_, number and weight average molecular mass, respectively; PDI = M_w_/M_n._

**Table 2:** Thermal properties determined from cooling and 2nd heating thermograms, storage modulus at 25 °C (from dynamic mechanical thermal analysis (DMTA)), and softening temperature (T_B_) in homopolymer PHF and series of PHF-*b*-F-pTHF copolymers.

Sample	T_g_ [°C]	ΔCp [J/g °C]	T_c_ [°C]	ΔH_c_ [J/g]	T_m_ [°C]	ΔH_m_ [J/g]	Xc [%]	E’ at 25 °C [MPa]	T_B_ [°C]
PHF	16.6	0.26	84	47.4	146	50.3	35.2	1373.3	143–152
PHF-*b*-F-pTHF 75/25	−36.4	0.28	79	40.8	141	35.1	24.5	310.5	134–145
PHF-*b*-F-pTHF 65/35	−58.6	0.33	72	39.7	137	36.6	25.6	232.2	129–143
PHF-*b*-F-pTHF 50/50	−65.5	0.38	54	28.8	127	27.6	19.3	131.2	115–135
PHF-*b*-F-pTHF 35/65	−65.8	0.43	47	22.9	115	20.4	14.1	77.7	98–119
PHF-*b*-F-pTHF 25/75	−66.6	0.58	27	16.5	102	11.4	7.9	35.2	90–105

T_g_, glass transition temperature; ΔC_p,_ change of heat capacity; T_c_, ΔH_c_, crystallization temperature and corresponding enthalpy of crystallization; T_m_, ΔH_m_, melting temperature and corresponding enthalpy of melting; Xc, degree of crystallinity, where ΔH_m_^0^ = 143 J/g [21]; E`, storage modulus at 25 °C; T_B_, the softening temperature range estimated using Boetius apparatus.

**Table 3:** Tensile properties of PHF-*b*-F-pTHF copolymers.

Sample	Hardness [Sh D]	E [MPa]	σ_y_ [MPa	ε_y_ [%]	σ_b_ [MPa]	ε_b_ [%]
PHF	68 ± 1	1134.9 ± 84.8	-	-	16.1 ± 4.2	1.01 ± 0.01
PHF-*b*-F-pTHF 75/25	51 ± 1	344.9 ± 69.1	16.4 ± 0.3	17.2 ± 1.7	43.8 ± 4.9	583.8 ± 46.1
PHF-*b*-F-pTHF 65/35	48 ± 1	232.7 ± 12.3	13.6 ± 0.3	18.0 ± 1.9	23.2 ± 0.7	541.3 ± 49.7
PHF-*b*-F-pTHF 50/50	41 ± 1	114.2 ± 13.4	-	-	21.8 ± 2.3	662.8 ± 65.5
PHF-*b*-F-pTHF 35/65	33 ± 1	70.0 ± 10.9	-	-	11.5 ± 0.1	665.0 ± 30.5
PHF-*b*-F-pTHF 25/75	26 ± 1	32.1 ± 7.6	-	-	7.2 ± 0.2	638.8 ± 33.1

E, Young’s modulus (calculated from strain 0.05% to 0.25%); σ_y_, ε_y_, tensile strength and elongation at yield, respectively; σ_b_, ε_b_, strength and elongation at break respectively.

**Table 4:** TGA data: temperatures of 5%, 10%, and 50% mass loss, the temperatures corresponding to the maximum of mass losses (T_DTG1_ and T_DTG2_), activation energies (E_a_) and correlation coefficient in linear regression (R) in an oxidizing and an inert atmosphere.

Sample	T_5%_ [°C]	T_10%_ [°C]	T_50%_ [°C]	T_DTG1_ [°C]	E_a_, (R) [kJ/mol]	T_DTG2_ [°C]
**Measurement in an oxidizing atmosphere**						
PHF	354	365	388	390	320.29 (0.9999)	500
PHF-*b*-F-pTHF 75/25	346	359	387	389	322.64 (0.9972)	505
PHF-*b*-F-pTHF 65/35	344	358	389	392	297.79 (0.9996)	506
PHF-*b*-F-pTHF 50/50	337	356	388	390	294.97 (0.9999)	504
PHF-*b*-F-pTHF 35/65	329	350	389	390	246.08 (0.9985)	504
PHF-*b*-F-pTHF 25/75	327	349	388	390	242.87 (0.9983)	507
**Measurement in an inert atmosphere**						
PHF	357	367	388	390	357.06 (1.0000)	-
PHF-*b*-F-pTHF 75/25	358	368	392	392	363.30 (0.9994)	-
PHF-*b*-F-pTHF 65/35	358	368	395	394	340.46 (0.9997)	-
PHF-*b*-F-pTHF 50/50	354	368	397	395	279.37 (0.9993)	-
PHF-*b*-F-pTHF 35/65	351	367	399	397	214.18 (0.9987)	-
PHF-*b*-F-pTHF 25/75	351	368	402	402	210.99 (0.9993)	-

## Data Availability

The data presented in this study are available on request from the corresponding author. The data are not publicly available due to the fact that these are data on the research work carried out in the project, which lasts until June 2022. And at the moment the data for this project is not kept in open repositories.
